# Deficient DNA base-excision repair in the forebrain leads to a sex-specific anxiety-like phenotype in mice

**DOI:** 10.1186/s12915-022-01377-1

**Published:** 2022-07-30

**Authors:** Flavia S. Mueller, René Amport, Tina Notter, Sina M. Schalbetter, Han-Yu Lin, Zuzana Garajova, Parisa Amini, Ulrike Weber-Stadlbauer, Enni Markkanen

**Affiliations:** 1 Institute of Veterinary Pharmacology and Toxicology, Vetsuisse Faculty, University of Zurich, 8057 Zurich, Switzerland; 2 Neuroscience Center Zurich, University of Zurich and ETH Zurich, Zurich, Switzerland; 3 Institute of Pharmacology and Toxicology, Faculty of Science, University of Zurich, 8057 Zurich, Switzerland

**Keywords:** Neuronal DNA damage, Anxiety, GABA, XRCC1, DNA repair

## Abstract

**Background:**

Neuropsychiatric disorders, such as schizophrenia (SZ) and autism spectrum disorder (ASD), are common, multi-factorial and multi-symptomatic disorders. Ample evidence implicates oxidative stress, deficient repair of oxidative DNA lesions and DNA damage in the development of these disorders. However, it remains unclear whether insufficient DNA repair and resulting DNA damage are causally connected to their aetiopathology, or if increased levels of DNA damage observed in patient tissues merely accumulate as a consequence of cellular dysfunction. To assess a potential causal role for deficient DNA repair in the development of these disorders, we behaviourally characterized a mouse model in which CaMKIIa-Cre-driven postnatal conditional knockout (KO) of the core base-excision repair (BER) protein XRCC1 leads to accumulation of unrepaired DNA damage in the forebrain.

**Results:**

CaMKIIa-Cre expression caused specific deletion of XRCC1 in the dorsal dentate gyrus (DG), CA1 and CA2 and the amygdala and led to increased DNA damage therein. While motor coordination, cognition and social behaviour remained unchanged, XRCC1 KO in the forebrain caused increased anxiety-like behaviour in males, but not females, as assessed by the light–dark box and open field tests. Conversely, in females but not males, XRCC1 KO caused an increase in learned fear-related behaviour in a cued (Pavlovian) fear conditioning test and a contextual fear extinction test. The relative density of the GABA(A) receptor alpha 5 subunit (GABRA5) was reduced in the amygdala and the dorsal CA1 in XRCC1 KO females, whereas male XRCC1 KO animals exhibited a significant reduction of GABRA5 density in the CA3. Finally, assessment of fast-spiking, parvalbumin-positive (PV) GABAergic interneurons revealed a significant increase in the density of PV+ cells in the DG of male XRCC1 KO mice, while females remained unchanged.

**Conclusions:**

Our results suggest that accumulation of unrepaired DNA damage in the forebrain alters the GABAergic neurotransmitter system and causes behavioural deficits in relation to innate and learned anxiety in a sex-dependent manner. Moreover, the data uncover a previously unappreciated connection between BER deficiency, unrepaired DNA damage in the hippocampus and a sex-specific anxiety-like phenotype with implications for the aetiology and therapy of neuropsychiatric disorders.

**Supplementary Information:**

The online version contains supplementary material available at 10.1186/s12915-022-01377-1.

## Background

DNA damage is associated with the molecular origin of many pathophysiological processes such as ageing, neurodegenerative disorders, neurodevelopmental disorders and cancer [1–3]. It can stem from exposure to exogenous DNA damaging agents (e.g. UV radiation, tobacco smoke) and from endogenous sources (e.g. oxidative stress) or it can be caused by a reduction in cellular DNA repair [4]. Base-excision repair (BER) is one of the most important DNA repair mechanisms responsible for correcting the frequently occurring small DNA base lesions and single-strand breaks (SSBs) [5]. BER is a highly coordinated process in which the scaffold protein X-ray repair cross-complementation group 1 (XRCC1) plays a critical function in stabilizing the two other core BER components DNA polymerase β and DNA ligase III [6, 7]. Therefore, a reduction or deletion of XRCC1 levels leads to a decrease in the overall BER capacity of a cell displayed by accumulation of persistent DNA damage and increase in genomic instability [5, 8, 9], which in turn increases the risk to develop cancer. While there is much evidence supporting a role for deficient DNA repair in the development of cancer [10], only a few studies have so far investigated the role of altered DNA repair in neurodevelopmental and neuropsychiatric disorders [2].

Neuropsychiatric disorders, such as schizophrenia (SZ), bipolar disorder (BD), major depressive disorder (MDD), autism spectrum disorder (ASD) or attention deficit hyperactivity disorder (ADHD), are cumulatively common, multi-factorial and multi-symptomatic disorders. They are characterized by impairments in cognitive function, emotions and behaviour and often associated with aberrant levels of anxiety and fear [11]. Despite decades of extensive research, neuropsychiatric disorders remain complex conditions with poorly defined neuropathology, and the underlying pathophysiological mechanisms are hardly understood [12]. Adding to this unsatisfactory situation, patients with neuropsychiatric disorders such as SZ or ASD often develop a variety of comorbidities, one example of which is cancer [13–15]. Some studies report a higher risk for specific cancers such as colon, breast and stomach cancers in patients with schizophrenia [14, 16]. In contrast, however, other studies have reported a significant reduction in overall cancer risk for patients with schizophrenia compared to the general population [17, 18]. Hence, the extent to which neuropsychiatric disorders such as schizophrenia are associated with altered cancer risk remains controversial [19, 20]. One explanation for these controversial findings may be the contribution of varying environmental factors such as smoking habits, nulliparity, obesity and exposure to antipsychotics [15, 20]. However, considering the fundamental role of DNA repair in cancer [21–23], it seems likely that altered expression of DNA repair genes and/or functional impairments in DNA repair mechanisms may also contribute to the observed altered cancer risk in patients with neuropsychiatric disorders. Indeed, several studies have shown that genetic polymorphisms in the core BER-protein XRCC1 or in XRCC1 interacting proteins such as 8-oxoguanine glycosylase (Ogg1) are implicated in patients with schizophrenia [24–27], ASD [28, 29] and bipolar disorder [30].

Against this background, we hypothesize that there is a pathological connection between altered DNA repair capacity and abnormal brain development relevant to neuropsychiatric disorders. To elucidate a possible role of the core BER-protein XRCC1 in regulating behaviour and cognition, we investigated animals that lack XRCC1 in the brain. As deletion of XRCC1 during early embryogenesis causes foetal lethality if applied in the entire body [31], or a defect in the genesis of cerebellar interneurons when using ablation during early embryonic development as with Nestin-Cre [32], we conditionally deleted XRCC1 in the forebrain during the postnatal period using CamKIIa-Cre. In mice, the expression of CamKIIa is largely restricted to the CNS and starts during early postnatal development, with peak expression obtained from neonatal periods onwards [33]. The regional and temporal expression of CamKIIa will thus lead to Cre-mediated deletion of floxed XRCC1 throughout major phases of brain development and maturation, thereby avoiding potential detrimental effects during embryonic development.

Applying this strategy, we investigated the effect of a lack of XRCC1 on various behavioural testing paradigms to assess anxiety-related (light–dark box test and open field test) and fear-related behaviour (context and cued Pavlovian fear conditioning), social interaction (Y-maze social interaction test), motor coordination (accelerating rotarod test) and cognitive functions (Y-maze spontaneous alternation and spatial recognition task). Based on our behavioural observations, we further examined GABAergic markers in key areas of the limbic system, which is well known to play an important role in regulating emotional and cognitive behaviours (for review, see e.g. [34]). Both, the amygdala and the hippocampus, represent key regions of the limbic system and are thought to mediate emotional reactions and to interact for translating emotion into particular behavioural outcomes [35]. Here, we focused on assessing the number of fast-spiking, parvalbumin-positive GABAergic interneurons and the alpha 5 and alpha 1 subunit of GABA(A) receptors (GABRA5 and GABRA1), based on their suggested pathophysiological relevance to neuropsychiatric anxiety disorders [36, 37]. Given that there are profound sex differences in the prevalence of a variety of psychiatric disorders and in related animal models [11, 38–42], we included both male and female animals.

Our results uncover a previously unappreciated connection between the DNA repair protein XRCC1, unrepaired DNA damage in the forebrain and a sex-specific anxiety-like phenotype.

## Results

### Conditional CaMKIIa-Cre-mediated knockout of XRCC1 leads to increased DNA damage in the forebrain

**Fig. 1:**
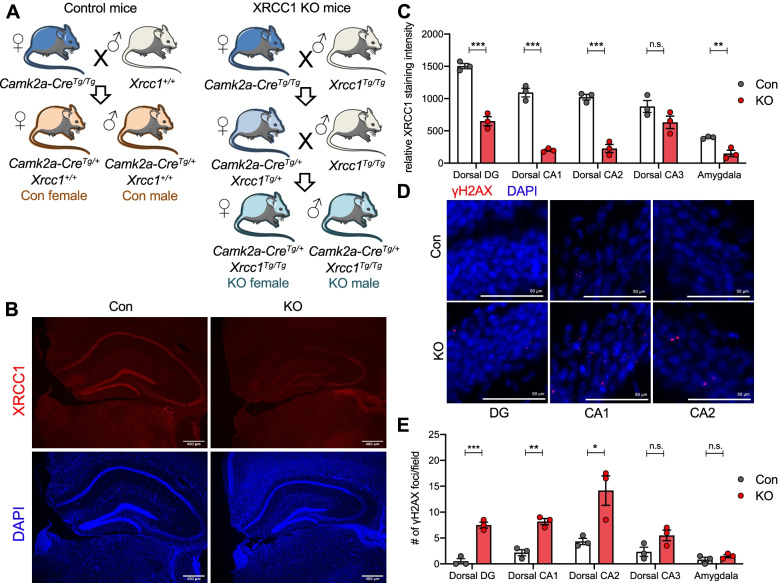
CamKIIa-Cre-mediated XRCC1 KO in different regions of the forebrain. **A** Breeding scheme used to obtain control and XRCC1 KO female and male animals. **B** Representative images showing staining of brain sections of control (left) and XRCC1 KO (right) male animals with XRCC1 antibodies (top) and Dapi (bottom) with a focus on the hippocampus. **C** Quantification of XRCC1 staining intensity as shown in **B** per area of interest in *n* = 3 per group. **p < 0.01 and ***p < 0.001 based on independent Student’s two-tailed *t*-test. Shown are all individual values, means ± SEM. **D** Representative images showing staining of brain sections of different hippocampal areas in control (top) and XRCC1 KO (bottom) male animals with γH2AX antibodies (red) and Dapi (blue). **E** Quantification of γH2AX staining intensity as shown in **D** per area of interest in *n* = 3 per group. *p < 0.05, **p < 0.01 and ***p < 0.001 based on independent Student’s two-tailed *t*-test. Shown are all individual values, means ± SEM

CaMKIIa-Cre drives efficient gene KO in postmitotic neurons in a highly restricted manner within the forebrain [43]. To obtain mice with a conditional knockout (KO) of XRCC1 starting during the early postnatal period in the forebrain, we applied a breeding strategy crossing XRCC1^Tg/Tg^ mice with CaMKIIa-Cre^Tg/Tg^ mice to generate animals of both sexes homozygous for XRCC1^Tg/Tg^ that express CaMKIIa-Cre (CaMKIIa-Cre^Tg/+^ XRCC1^Tg/Tg^, henceforth referred to as “KO”) (Fig. 1A). Mice heterozygous for CaMKIIa-Cre and a wildtype XRCC1 locus were used as control (CaMKIIa-Cre^Tg/+^ XRCC1^+/+^).

Immunohistochemical staining of XRCC1 in forebrain regions of adult mice clearly revealed a strong decrease in XRCC1 expression in the dorsal DG, CA1 and CA2 subregions of the hippocampus, as well as in the amygdala of KO animals compared to controls, but not in the dorsal CA3 in both male and female animals (Fig. 1B and C and Additional file 1: Fig. S1A and S1B). The corresponding ventral structures showed a slight reduction in XRCC1 levels that failed to reach significance (Fig. S1C). These observations are in line with published data on the CaMKIIa-Cre model [43] and our findings on a CaMKIIa-Cre–td-Tomato mouse showing highest expression of CaMKIIa-Cre in CA1, CA2 and DG, with only minor expression in CA3 (data not shown). Thus, we conclude that CaMKIIa-Cre-mediated KO mainly deletes XRCC1 in the dorsal DG, CA1 and CA2 and in the amygdala.

KO of XRCC1 is expected to induce persistent DNA damage through the incapacitation of BER [32]. To validate that the CaMKIIa-Cre-mediated deletion of XRCC1 had a functional relevance, we assessed the amount of DNA damage in the affected forebrain structures using immunohistochemical detection of the DNA damage marker γH2AX [32]. As expected, there was a significant increase of γH2AX foci/field in the dorsal DG, CA1 and CA2 in KO animals (Fig. 1D and E and Fig. S1D). As expected from the lack of effect on XRCC1 levels in this region, the number of γH2AX foci did not change significantly in dorsal CA3 and amygdala of KO compared to control animals. In summary, these results demonstrate that the KO of XRCC1 in dorsal DG, CA1 and CA2 indeed leads to the formation of increased DNA damage in these regions, further validating our experimental approach.

### CaMKIIa-Cre-driven XRCC1 KO does not cause changes in motor coordination, cognition or social behaviour

**Fig. 2:**
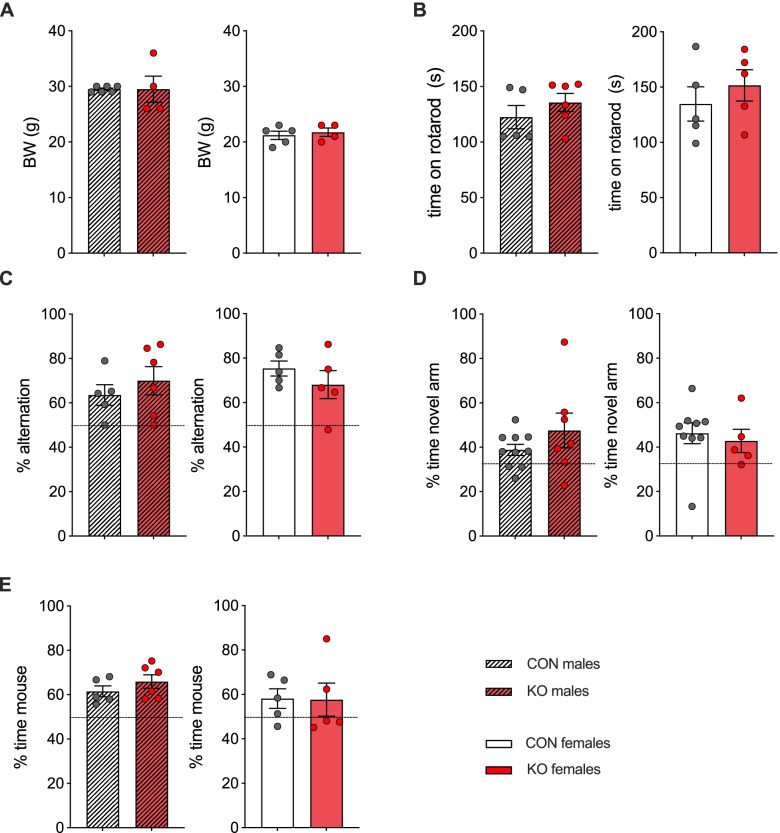
CaMKIIa-Cre-driven XRCC1 KO does not cause changes in motor coordination, cognition or social behaviour. **A** Body weight (BW) of animals measured after testing at 17 weeks of age. **B** Accelerating rotarod test, showing time the animals stayed on the rotarod in s. **C** Spontaneous alternation test. The bar plots depict the percentage of alternation in the Y-maze for male (left) and female (right) XRCC1-KO and control mice. The dashed line indicates chance level (50%). **D** Y-maze spatial recognition test. The bar plots show the percent (%) time spent in the novel arm during the choice phase of the spatial working memory test in the Y-maze for male (left) and female (right) XRCC1-KO and control mice. The dashed line indicates chance level (33%). **E** Social interaction test. The bar plots depict the percent (%) time spent with an unfamiliar mouse (compared to an inanimate dummy) for both sex and groups. The dashed line indicates chance level (50%). **A** *N* = 6 Con and 4 KO males, 5 Con and 4 KO females; **B**, **C**, **E** *N* = 5 Con and 6 KO males, 5 Con and 5 KO females; **D** *N* = 10 Con and 7 KO males, 9 Con and 5 KO females. Shown are all individual values, means ± SEM

Nestin-Cre-mediated deletion of XRCC1 in the brain during early embryonic development has been reported to cause profound neuropathology that is characterized by mild ataxia accompanied by episodic spasms and seizures due to the loss of cerebellar interneurons and a 25% smaller body weight compared to controls [32, 44, 45]. In contrast to the Nestin-Cre model, we could not observe any overt abnormalities in body weight (Fig. 2A), gait or other obvious phenotypic features caused by CaMKIIa-Cre-mediated KO of XRCC1 in the forebrain. To assess motor coordination, we performed an accelerating rotarod test. In keeping with our phenotypical observation, the KO animals did not show any change in motor coordination compared to controls (Fig. 2B). Thus, CaMKIIa-Cre-mediated ablation of XRCC1 in the forebrain does not seem to cause the profound and obvious pathologies in neural development that are seen in the Nestin-Cre model, suggesting they are suitable models to assess more subtle effects of unrepaired DNA damage on behavioural phenotypes.

We further assessed the effect of XRCC1 KO on cognitive performance including tests for spatial recognition memory and for short-term working memory. The genetic manipulation of XRCC1 did not affect working memory function in the spontaneous alternation test in either male or female mice (Fig. 2C). The genetic manipulation did also not affect the preference for the novel arm in the Y-maze test of spatial recognition memory in male or female animals (Fig. 2D).

We also assessed sociability in the social interaction test in which mice were allowed to explore an unfamiliar peer mouse or an inanimate dummy object. XRCC1 KO did not affect social behaviour, neither in male nor in female animals (Fig. 2E). These results were not confounded by differences in general locomotor activity, as indexed by the total distance moved during the tests, which was highly comparable between groups (Fig. S2A, B and C). Hence, CaMKIIa-Cre-mediated ablation of XRCC1 in the forebrain did not affect social interaction or cognitive performance.

### Innate anxiety-related behaviour in XRCC1 knockout mice

**Fig. 3:**
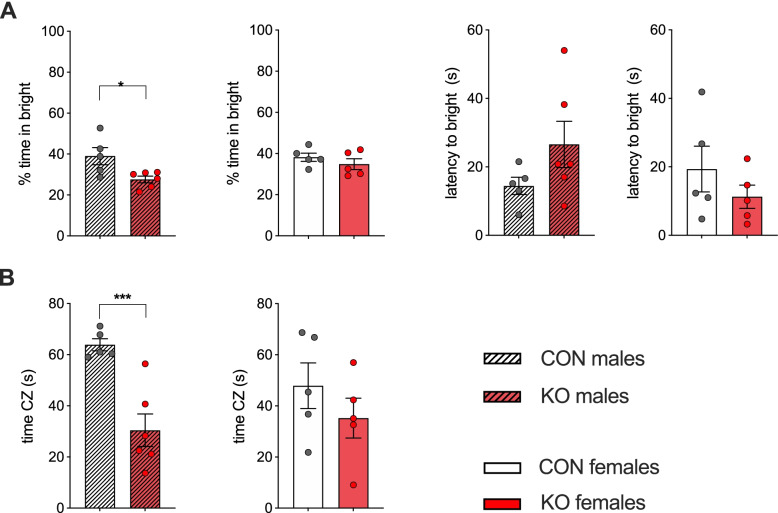
Innate anxiety-related behaviour in CamKIIa-Cre XRCC1 KO mice. **A** Light–dark box test. The bar plots depict percent time spent in the bright compartment compared to the dark compartment and the latency to enter the bright compartment (s) for both male and female animals (left and right bar plot, respectively). **B** Open field test. Time spent (s) in the centre zone of the open field by male (left bar plot) and female (right bar plot) XRCC1-KO and control mice. *N* = 5 Con and 6 KO males, 5 Con and 5 KO females. Shown are all individual values, means ± SEM

To investigate the effect of increased DNA damage caused by XRCC1 KO in the forebrain on affective behaviours, we evaluated the impact of XRCC1 KO on measures of innate anxiety-like behaviour using the light–dark box (LDB) and the open field tests. Male KO mice displayed increased anxiety-like behaviour in the light–dark box test, as evident by the reduced time spent in the bright compartment as compared to control animals (*t*_(9)_ = 2.71, *p* < 0.05; Fig. 3A). In support of this notion, male KO mice also showed a trend towards an increased latency to enter the bright compartment of the testing apparatus, albeit this trend did not reach statistical significance (*t*_(9)_ = 1.55, *p* = 0.15) (Fig. 3A). We observed no difference in the behaviour of female KO mice compared to female controls in the LDB test, neither in the percent time spent in nor in the latency to enter the bright compartment (Fig. 3A). The assessment of the innate anxiety-like behaviour in the LDB was not confounded by differences in locomotor activity, as the distance moved during the test was similar in both groups for male and female animals (Fig. S2D). In line with the results of the LDB test, male KO mice showed increased innate anxiety-like behaviour in the open field test as evident by the reduced time spent in the centre zone of the open field (*t*_(9)_ = 4.54, *p* < 0.01; Fig. 3B). Again, female KOs did not display any differences in innate anxiety-like behaviour function in the open field test (Fig. 3B). The open field test was not confounded by differences in general locomotor activity levels (Fig. S2E). Concluding, these results suggest that XRCC1 KO in the forebrain induces an increase in anxiety-related behaviour in male, but not female animals.

### Learned fear-related behaviour in XRCC1 KO mice

**Fig. 4:**
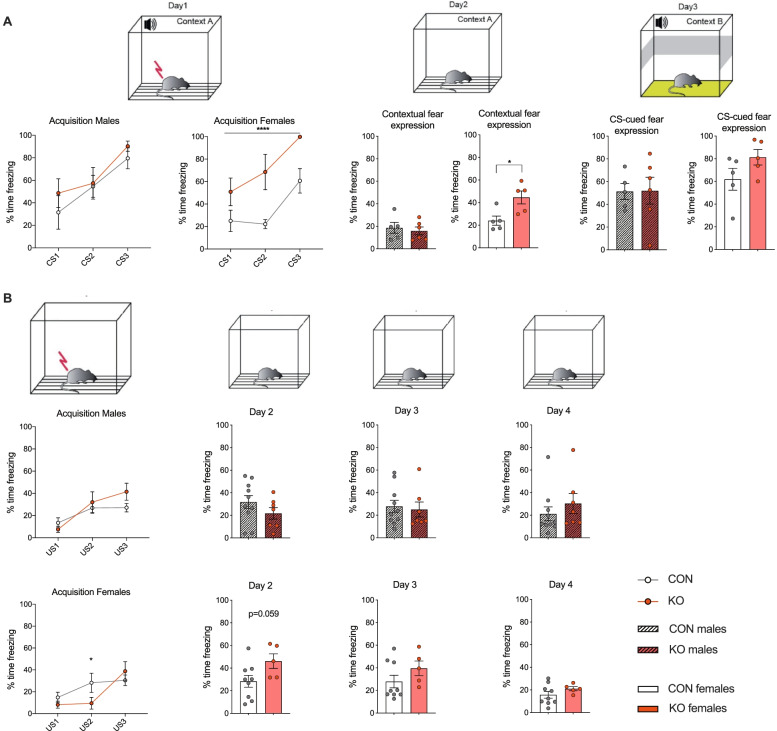
Learned fear-related behaviour in CamKIIa-Cre XRCC1 KO mice. **A** Cued Pavlovian fear conditioning. The line plots show the fear response (indexed by percent time freezing) acquired to three successive presentations of the conditioned stimulus (CS) (tone) followed by the unconditioned stimulus (US) (foot shock) for male and female animals. The bar plots depict the expression of conditioned fear towards the context (*contextual fear expression)* during the context test and the expression of auditory-cued conditioned fear during the tone-CS test (*CS-cued fear expression*) as measured by percentage time freezing. *N* = 5 Con and 6 KO males, 5 Con and 5 KO females. Shown are all individual values, means ± SEM. ****p < 0.001 based on repeated-measures ANOVA, *p < 0.05 based on independent Student’s two-tailed *t*-test. **B** Acquisition, expression and extinction of contextual fear. The line plots show percent time freezing acquired following three successive presentations of foot shocks (unconditioned stimulus, US). The bar plots represent percent time freezing during the fear expression (day 2) and extinction (day 3 and day 4) phases. *N* = 10 Con and 7 KO males, 9 Con and 5 KO females. Shown are all individual values, means ± SEM. *p < 0.05, based on post hoc comparisons following repeated-measures ANOVA

To test whether a forebrain-specific XRCC1 KO may also modulate conditioned forms of anxiety/fear, we compared KO and control animals in the cued (Pavlovian) fear conditioning test and the contextual fear extinction test. The cued Pavlovian fear conditioning involves a tone as a conditioned stimulus (CS) and electric foot shock as the unconditioned stimulus (US). In the acquisition of conditioned fear during successive CS-US trials, female KO mice displayed increased freezing in the initial acquisition of the conditioned fear response compared to control animals. While the amount of percentage time freezing during conditioning increased in both groups as a function of CS-US trials (main effect of trials: *F*_(2,16)_ = 7.925, *p* < 0.05), indicating intact acquisition of the conditioned fear response to the auditory CS in both groups, the level of freezing was significantly increased in female KO animals compared to controls (main effect of genotype: *F*_(1,8)_ = 38.12, *p* < 0.0001; Fig. 4A). In addition, female KO mice also showed increased conditioned fear towards the context (placed in the same context but without the US and CS present) (*t*_(8)_ = 2.93, *p* < 0.05). Interestingly, the expression of auditory-cued conditioned fear during the tone-CS test did not differ significantly between female KO and control animals, indicating an increased association of the conditioned fear with the context, rather than the tone, in the KO females. In contrast to the findings in female animals, we did not observe learned fear-related abnormalities in male KO mice compared to controls, neither in the acquisition phase nor in the fear towards the context or the auditory-cued conditioned fear (Fig. 4A). The observed effects were not confounded by differences in general freezing levels, as indexed by highly comparable freezing levels during the initial habituation phase (Fig. S2F).

In addition, we performed the contextual fear extinction test in an independent cohort of animals (see Additional file 1: Table S1). For this test, electric foot shocks (US) are used to promote the formation of fear memory to a specific context followed by assessing contextual fear expression and extinction for 3 days. The acquisition of fear conditioning towards the context was intact in both, male and female KO and control mice, as indexed by an increase in percent time freezing during conditioning as a function of trials (main effect of trials: males *F*_(2,30)_ = 11.69, *p* < 0.001; females *F*_(2,24)_ = 12.77, *p* < 0.001). KO females displayed a slight attenuation of the development of the context-conditioned freezing response, which was notable in the second conditioning trial, as supported by a significant interaction of trials and genotype (*F*_(2,24)_ = 4.1, *p* < 0.05; Fig. 4B). On the following day (day 2), when the animals were placed in the identical context but without receiving any further electric foot shock, female KO mice showed a trend towards increased contextual fear response compared to control animals (*t*_(12)_ = 2.09, *p* = 0.059), whereas male KOs did not show a significant difference. On day 3 and 4, the conditioned contextual fear response was highly comparable for both groups in males and females (Fig. 4B, top and bottom panels, respectively). Again, the effects on contextual fear were not confounded by differences in basal freezing, as indexed by highly comparable freezing levels during the initial habituation phase (Fig. S2G).

As such, these results uncover an increased expression of learned fear-related behaviours in female, but not male animals, with an XRCC1 KO in the forebrain.

### Effects of XRCC1 knockout on GABAergic markers in the brain

**Fig. 5:**
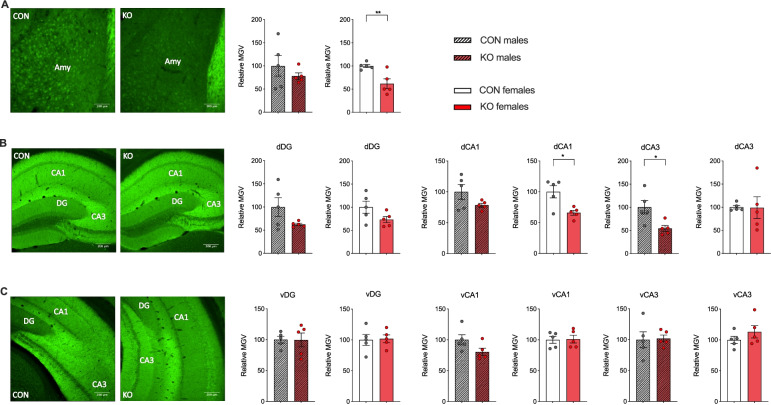
GABRA5 receptor density in XRCC1 KO mice. The images show representative coronal brain sections of female control and XRCC1 KO animals stained with anti-GABRA5 antibody at the level of **A** the amygdala, **B** the dorsal hippocampus and **C** the ventral hippocampus. The bar plots depict the relative mean grey value (MGV) of GABRA5 in **A** the amygdala (Amy), **B** the dorsal and **C** ventral DG, CA1 and CA3 (dDG, dCA1, dCA3 and vDG, vCA1, vCA3, respectively) of male and female XRCC1-KO and control mice. *N* = 5 Con and 5 KO males, 5 Con and 5 KO females. Shown are all individual values, means ± SEM. *p < 0.05 and **p < 0.01 based on independent Student’s two-tailed *t*-test

In view of our behavioural findings in the XRCC1 KO mice, we examined GABAergic markers in the amygdala and in the hippocampus, both of which regions are believed to critically modulate both innate anxiety and learned fear [46, 47]. We thereby focused on assessing the number of a specific subpopulation of GABAergic interneurons and of distinct alpha subunits of GABA(A) receptors. Parvalbumin is mainly expressed by fast-spiking GABAergic interneurons and is pivotal for proper neuronal synchronization by providing inhibitory input to axon initial segments [48]. In addition, we examined distinct alpha subunits of the GABA(A) receptor (GABRA5 and GABRA1), based on their pathophysiological relevance to neuropsychiatric anxiety disorders [36, 37]. The relative density of GABRA5 in the amygdala was reduced in female KO mice relative to control mice (*t*_(8)_ = 3.46, *p* < 0.01; Fig. 5A). In addition, female KOs displayed significantly reduced GABRA5 receptor density in the CA1 subregion of the dorsal hippocampus relative to control animals (*t*_(8)_ = 3.19, *p* < 0.05), while there were no significant changes in GABRA5 receptor density in other subregions of the dorsal hippocampus (Fig. 5B) and also not in the ventral hippocampus (Fig. 5C). Similar region-specific findings were obtained with regard to GABRA5 receptor density in male KO mice. While male KO mice exhibited a significant reduction of GABRA5 receptor density in the CA3 subregion of the dorsal hippocampus relative to control animals (*t*_(8)_ = 2.90, *p* < 0.05) (Fig. 5B), no significant changes were observed in other subregions of the dorsal hippocampus (Fig. 5B), in the ventral hippocampus (Fig. 5C) or the amygdala (Fig. 5A and Fig. S3). Similar patterns were found for the relative density of GABRA1, with reduced GABRA1 density in the amygdala of female KO mice relative to control mice, while male KO mice exhibited a significant reduction of GABRA1 receptor density in the CA3 subregion of the dorsal hippocampus relative to controls (see Fig. S4 and S5).

**Fig. 6:**
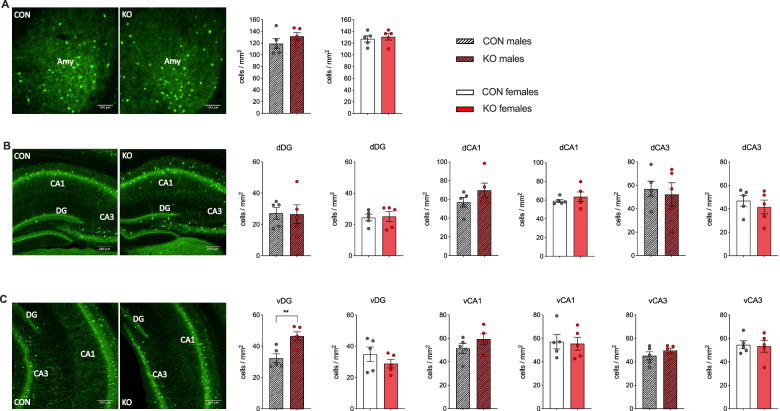
Parvalbumin cell numbers in CamKIIa-Cre XRCC1 KO mice. The pictures show representative sections of female control and XRCC1 KO animals stained with anti-parvalbumin antibody in **A** amygdala, **B** dorsal hippocampus and **C** ventral hippocampus. The bar plots depict the number of parvalbumin-positive (PV+) cells per mm^2^ in **A** the amygdala (Amy), **B** the dorsal and **C** ventral DG, CA1 and CA3 (dDG, dCA1, dCA3 and vDG, vCA1, vCA3, respectively) of XRCC1-KO and control mice. *N* = 5 Con and 5 KO males, 5 Con and 5 KO females. Shown are all individual values, means ± SEM. **p<0.01 based on independent Student’s two-tailed *t*-test

In addition to GABRA5 and GABRA1 receptor density, we assessed the number of parvalbumin-positive, GABAergic interneurons in KO and control animals. The number of parvalbumin-positive (PV+) cells was not changed in the amygdala (Fig. 6A) or the dorsal (Fig. 6B) hippocampus of male and female KO mice compared to control mice. However, male KOs showed a significant increase of PV+ cells per mm^2^ in the DG subregion of the ventral hippocampus (*t*_8_ = 3.53, *p* < 0.01; Fig. 6C) compared to control animals, while female KO mice did not show changes in PV+ cells in the ventral hippocampus. All results have been summarized in supplementary tables S2 and S3.

## Discussion

Using a novel and selective model of XRCC1 KO in mice, our study is the first to demonstrate a role of unrepaired DNA damage in the forebrain in the modulation of innate anxiety and learned fear in a sex-dependent manner. Male animals consistently showed increased anxiety-like behaviour in two behavioural tests, commonly used in preclinical models of anxiety disorders [49, 50]. On the other hand, female mice displayed increased expression of conditioned auditory-cued and contextual fear, which was characterized primarily by an increased expression of learned fear towards the context. These behavioural manifestations were accompanied by region-specific changes of GABAergic markers in key areas of the limbic system, suggesting that an increase in unrepaired DNA damage by XRCC1 KO can induce GABAergic abnormalities in brain regions, commonly affected in neuropsychiatric anxiety disorders [51–54].

In contrast to our results with CaMKII-Cre-driven XRCC1 KO, Nestin-Cre-mediated KO of neuronal XRCC1 has been shown to cause profound neuropathology characterized by mild ataxia, episodic spasms and seizures due to the loss of cerebellar interneurons and a 25% smaller body weight compared to controls [32, 44, 45]. We hypothesize that the milder phenotype we observe (neither a change in body weight nor spasms/seizures and normal motor coordination) using CaMKIIa-Cre-mediated ablation of XRCC1 in the forebrain derives from differences in the time of onset of Cre expression and XRCC1 deletion, as well as locoregional differences in expression between the two models. While Nestin-Cre expression is observed already during early embryonic development from day 8 onwards in large parts of the developing central nervous system [55], CaMKIIa-Cre expression has been documented to start during the early postnatal period and to be restricted to mainly the forebrain regions CA1–CA3 [43]. Hence, the much stronger phenotype seen using the Nestin-Cre model might also, in part, be attributed to changes in prenatal brain development. The more subtle changes observed in the CaMKIIa-Cre model suggest this approach to be suitable to assess effects of unrepaired DNA damage on behavioural phenotypes. In the Nestin-Cre model, the pathology is driven through hyperactivity of the DNA strand break sensor PARP1 through deregulation of presynaptic calcium signalling and can be rescued by inhibition or deletion of PARP1. Whether the same signalling pathways are involved in the CaMKIIa-Cre animals’ phenotype remains an open question, which should be addressed in future studies.

### Sex-specific effects on innate anxiety and learned fear

The sex-dependent differential effects on innate anxiety and learned fear might at first seem counterintuitive, if not contradictory. However, there are a number of important distinctions between these behavioural domains, especially with respect to the underlying neuropsychological mechanisms. Innate anxiety is typically defined as a state of goal conflict or uncertainty that does not require any associative learning process for its manifestation [56, 57]. On the other hand, the development of conditioned fear is dependent on a learning process, as the subject needs to associate an initially neutral stimulus or context with an aversive consequential event [56, 57]. Hence, as opposed to innate anxiety, conditioned fear is not driven by a state of uncertainty or goal conflicts, but rather by prediction and contingency [58]. Along this line, also the neuropsychological processes involved in conditioned fear and anxiety are viewed as distinct from each other [58]. Our data highlight that both of these neuropsychological domains are sensitive to and affected by increased neuronal DNA damage, but in a sex-specific manner. These results emphasize the importance of assessing phenotypes in a sex-specific manner, similarly to what has been proposed for epidemiologic investigations assessing anxiety- and fear-related disorders [38–41].

Interestingly, the behavioural effects were specific for anxiety- and fear-related paradigms, as we did not observe a difference in other behavioural domains, such as social behaviour or working memory.

### GABAergic changes induced by XRCC1KO

Within the CNS, limbic circuitries are believed to critically modulate both innate anxiety and learned fear [46, 47]. Within the limbic areas investigated, we found that XRCC1 KO caused significant, sex-dependent GABAergic alterations in the amygdala and distinct subregions of the hippocampus. Neuronal dysfunctions in the limbic GABAergic system have been widely documented for their involvement in neuropsychiatric disorders, including anxiety-related diseases (for review, see e.g. [59]). More specifically, there is accumulating evidence for the limbic GABAergic system being critical for regulating innate and conditioned forms of anxiety and fear [47, 60–62].

The emergence of altered PV^+^ cell number in the ventral DG is in line with the growing literature suggesting that the ventral part of the hippocampus is more vulnerable than the dorsal part with respect to PV abnormalities following exposure to stressors, such as e.g. oxidative stress during development [63]. Along this line, there is evidence for an involvement of PV^+^ interneurons in the ventral DG affecting innate anxiety, while not affecting contextual fear memory [64]. However, the upregulation of PV^+^ cells in male animals, along with displaying higher levels of innate anxiety, seems contradictory at first, as the majority of literature suggests a reduction in PV cells being associated with higher levels of anxiety [64–66]. It is important to note, though, that the implication of parvalbumin in anxiety-like behaviour seems to be highly region- and sex-specific: as such, there is also evidence for an anxiogenic effect of PV^+^ cell activation [67, 68].

Relatedly, our study revealed that XRCC1 KO significantly reduced the expression of subunits of the GABA_A_ receptor in a region- and sex-specific manner. As such, we observed decreased GABRA5 and GABRA1 in the amygdala of female animals and of GABRA5 in the dorsal CA1 and CA3 subregions of female and male animals, respectively. Reduced levels of GABA(A) receptors have been repeatedly implicated in fear regulation and anxiety [69, 70], along with indices for a decline in GABA(A) receptor-binding sites in the mesolimbic system of patients suffering from anxiety disorders [71], which is in line with our findings in the XRCC1 KO mouse model. Given that the effects were found to be sex-specific, our findings add to the growing evidence that a specific genetic manipulation can result in distinct molecular adaptations and phenotypes in males and females [72–75] and highlight the critical importance of including both sexes in the exploration of the presented mouse model. Additional studies are warranted in order to identify the underlying mechanisms driving the emergence of sex-dependent molecular and behavioural consequences of XRCC1 KO.

### Possible links between DNA damage, GABAergic abnormalities and behaviour

The GABAergic changes emerging in the limbic system of XRCC1 KO mice likely represent secondary responses to primary changes in upstream neuronal signalling pathways that are directly affected by accumulating DNA damage. Hence, we do not know at the present stage whether the numeric changes in PV cells and GABA_A_ receptor subunits are accounted for by altered gene transcription and subsequent protein synthesis, or, alternatively, whether these effects can be explained by actions on neurogenesis and/or (programmed) cell death, so that future investigations towards this direction are therefore clearly warranted. In this context, it has been shown that mice lacking the DNA glycosylases OGG1 and MUTYH — upstream enzymes involved in BER responsible for removing damaged bases — show a decrease in anxiety and impaired learning ability, potentially due to altered hippocampal gene expression [76]. Moreover, the knockout of DNA glycosylases NEIL1 and NEIL2 causes dysregulation of genes that are relevant for synaptic function in CA1 and leads to reduced anxiety and improved learning in these animals that is potentially linked to changes in GABAergic signalling [77].

Future studies will also be needed to explore the contribution of these GABAergic changes to behavioural dysfunctions. We acknowledge that our study falls short in dissecting the relative contribution of specific GABAergic abnormalities to the emergence of the observed anxiety- and fear-related phenotypes. Indeed, even though the association between the DNA damage-induced behavioural impairments and GABAergic alterations is intriguing, we did not further attempt to delineate the functional contribution of GABAergic abnormalities to the induction of the behavioural impairments, so these relationships remain descriptive and speculative at present. Additional work will be needed to further address this issue using pharmacological and/or genetic approaches, which could serve to mitigate the behavioural deficits by targeting distinct GABAergic abnormalities. On speculative grounds, however, a functional association seems reasonable, as both the amygdala and hippocampus play prominent roles in innate anxiety and the acquisition and expression of contextual fear responses, respectively [46, 47, 51–53, 78]. Since XRCC1 KO mice displayed significant alterations of GABAergic markers in amygdala and hippocampus, these may in turn influence regional activity and ultimately alter the expression of innate or learned fear [79–81].

## Conclusions

Our study highlights a novel role of unrepaired DNA damage in the regulation of emotional behaviour. In light of the clinical availability of inhibitors that attenuate aberrant signalling activity resulting from unrepaired DNA damage, it will be interesting to address whether these compounds could have a role in the treatment of the pathophysiology of anxiety disorders and post-traumatic stress disorder. Future investigations of the possible link between XRCC1, DNA damage and innate anxiety and fear will help to gain more insight into the intricate mechanisms whereby accumulating DNA damage can influence complex brain and behavioural functions.

## Methods

### Animals

#### Breeding

The C57BL/6 XRCC1 loxP/loxP mouse line (XRCC1^Tg/Tg^) was originally generated by Peter J. McKinnon (St. Jude’s hospital, Memphis TN, USA) and transferred to our animal facility by embryotransfer [32]. To maintain this strain, animals were regularly backcrossed on C57BL/6J control mice. C57BL/6J Cre-CamKIIa (JAX stock #005359; CamKIIa-Cre^Tg/Tg^) mice and the appropriate C57BL/6J control mice (JAX stock #000664; XRCC1^+/+^) were purchased from Charles River Germany. According to recommendations by the provider, CamKIIa-Cre^Tg/Tg^ were maintained as homozygous colony. To produce the experimental KO animals, CamKIIa-Cre^Tg/Tg^ females were crossed to XRCC1^Tg/Tg^ males to obtain CamKIIa-Cre^Tg/+^ XRCC1^Tg/+^ females. These females were further crossed to XRCC1^Tg/Tg^ males to obtain both male and female CamKIIa-Cre^Tg/+^ XRCC1^Tg/Tg^ animals (KO females and KO males). Control male and female CamKIIa-Cre^Tg/+^ XRCC1^+/+^ animals were obtained by crossing CamKIIa-Cre^Tg/Tg^ females to XRCC1^+/+^ males (Fig. 1). This breeding approach was based on the fact that the CaMKIIa Cre transgene had significant effects on behaviour as compared to WT C57BL/6J mice (Fig. S6). Hence, we only used CamKIIa-Cre^Tg/+^ XRCC1^+/+^ as controls in subsequent experiments, so as to control for the effect of the CaMKIIa-Cre transgene and avoid false positive interpretations (more conservative approach). At postnatal day (PND21), littermates of the same sex were weaned and caged separately in groups of 3–5 animals per cage. During weaning, ear punch biopsies were collected for polymerase chain reaction (PCR)-based genotyping. After confirmation of the genotype, the animals were rehoused according to their genotype.

The animals were kept in a specific-pathogen-free (SPF) holding room, which was temperature- and humidity-controlled (21 ± 3 °C, 50 ± 10%) and kept under a reversed light–dark cycle (lights off: 09:00 AM–09.00 PM). All animals had ad libitum access to the same food (Kliba 3336, Kaiseraugst, Switzerland) and water throughout the entire study. All procedures had been previously approved by the Cantonal Veterinarian’s Office of Zurich, and all efforts were made to minimize the number of animals used and their suffering.

#### Genotyping

For initial genotyping, genomic DNA from ear biopsies was extracted by heating samples in 75 μl of lysis buffer (25 mM NaOH, 0.2 mM EDTA in dH2O) at 95°C for 20 min. After this, 75 μl of neutralization buffer (40 mM Tris-HCl pH 6.5) was added, and the supernatant was diluted 1:10 in dH2O. 2.5 μl of this solution containing genomic DNA was used for subsequent PCR. PCR to determine the XRCC1 allele was performed as described in [32]. To detect the presence of CaMKIIa-Cre, the primers (fw: 5′-GCGGTCTGGCAGTAAAAACTATC-3′, rev: 5′-GTGAAACAGCATTGCTGTCACTT-3′) were used as suggested by the vendor of the animals (The Jackson Laboratories). To confirm correct genotyping, genotyping was repeated in all animals after euthanasia.

### Behavioural testing

Three cohorts of animals were produced (Additional file 1: Table S1) to perform behavioural testing (see below). The behavioural testing started when the offspring reached 12 weeks of age and included tests for locomotor function, working and spatial recognition memory and social behaviour, as well as for anxiety and learned fear-related behaviour as detailed below. The behavioural testing was following the same testing order for each offspring within a specific cohort and included a test-free resting period of 3–4 days in between each test.

#### Locomotion (rotarod test)

Motor coordination was assessed using a rotarod with a diameter of 3 cm (Ugo Basile, Gemonio, Italy). To start a trial, each mouse was gently placed onto the rotarod. The rotarod started with 4 rpm and accelerated within 180 s to 40 rpm. A trial was stopped when the animals fell off the rotarod onto the landing platform or after 210 s. The latency to fall down was measured over 3 trials.

#### Working and spatial recognition memory test

##### Working memory test

A spontaneous alternation task in the Y-maze was used to assess working memory [82]. This task is based on the innate tendency of rodents to explore novel environments, that is, their preference to investigate a new arm of the maze rather than returning to one that was previously visited.

The apparatus and testing procedures were validated before and described in detail elsewhere [83]. In brief, the Y-maze was made of transparent Plexiglas and consisted of three identical arms (50 cm × 9 cm; length × width) surrounded by 10-cm high Plexiglas walls. The three arms radiated from a central triangle (8 cm on each side) and spaced 120° from each other. A digital camera was mounted above the Y-maze apparatus and images were transmitted to a PC running the EthoVision tracking system (Noldus Information Technology), which calculated the total distance moved into the three arms and the centre zone of the Y-maze.

To begin a trial, the animals were placed in the centre of the Y-maze and allowed to freely explore the maze for 5 min. An observer who was blind to the treatment conditions viewed the mice through a distant video recording system and recorded the number and sequence of arm entries (defined as entry of the whole body into an arm) during the 5-min testing period. Alternation was defined as entry into the three arms in any non-repeating order (for example, ABC, BAC, CBA). The percentage alternation was calculated as the total number of alternations divided by the possible alternations given by the number of arm entries (total number of arm entries: 2). In addition to the analysis of percentage alternation, the total distance moved was recorded and analysed to assess general exploratory activity during the 5-min test period.

##### Spatial recognition memory

Short-term spatial recognition memory was assessed using a Y-maze test as established and validated before [84]. The Y-maze apparatus is the same as used for the spontaneous alternation task described above. The test consisted of two phases, the so-called sample and choice phases. The allocation of arms (start, familiar and novel arm) to a specific spatial location was counterbalanced across the experimental conditions.

Sample phase: The animals were allowed to explore two arms (referred to as “start arm” and “familiar arm”). Access to the remaining arm (“novel arm”) was blocked by an opaque barrier wall. To begin a trial, the animal was introduced at the end of the start arm and was allowed to freely explore both the start and the familiar arms for 5 min. The animal was then removed and kept in a holding cage prior to commencement of the choice phase. The barrier door was removed, and the maze was cleaned with water and dried thereafter to avoid olfactory cues.

Choice phase: The animal was introduced to the maze following a retention interval of 1 min. During the choice phase, the barrier wall was removed so that the animals could freely explore all three arms of the maze for 5 min. On each trial, the time spent in each of the three arms was recorded. The relative time spent in the novel arm during the choice phase was calculated by the formula ([time spent in the novel arm]/[time spent in all arms] × 100) and used as the index for short-term spatial recognition memory. In addition, total distance moved on the entire maze was recorded and analysed in order to assess general locomotor activity.

#### Social behaviour

Social interaction was assessed by analysing the relative exploration time between an unfamiliar congenic mouse and an inanimate dummy object using methods established before [83, 85]. Two out of the three arms of the Y-maze (apparatus described in the section “Social behaviour”) contained a rectangular wire grid cage (13 cm × 8 cm × 10 cm, length × width × height; bars horizontally and vertically spaced 9 mm apart). The third arm did not contain a metal wire cage and served as the start zone (see below).

All animals were first habituated to the test apparatus on the day before social interaction testing. This served to familiarize the test animals with the apparatus and to reduce novelty-related locomotor hyperactivity, which may potentially confound social interaction during the critical test phase. During habituation, each test mouse was gently placed in the start arm and allowed to explore the apparatus for 5 min.

The test phase took place 1 day after the habituation day. During the test phase, one metal wire cage contained an unfamiliar C57Bl6 mouse of the same sex (10–12 weeks of age), whereas the other wire cage contained an inanimate dummy object. The latter was a black scrunchie made of velvet material. The allocation of the unfamiliar live mouse and inanimate dummy object to the two wire cages was counterbalanced across experimental groups. To start a test trial, the test mouse was gently placed in the start arm and allowed to explore freely for 5 min. Behavioural observations were made by an experimenter blind to the experimental conditions, and social interaction was defined as nose contact within a 5-cm interaction zone. The relative time spent with the live mouse was calculated by the formula ([time spent with the mouse]/[time spent with the inanimate object + time spent with the mouse]) × 100 and used to compare the relative exploration time between the unfamiliar mouse and the inanimate dummy object. The total distance moved during the test was also measured to analyse general exploratory activity. This was achieved by a digital camera mounted above the apparatus, which provided images that were transmitted to a PC running the EthoVision tracking system (Noldus Information Technology, The Netherlands).

#### Anxiety-like behaviour

##### Light–dark box test

Innate anxiety-like behaviour was assessed using the light–dark box (LDB) test. The LDB test is based on the natural aversion of mice to brightly illuminated areas and on their spontaneous exploratory behaviour in novel environments [86]. The LDB apparatus consisted of two-way shuttle boxes (30 × 30 × 24 cm; Multi Conditioning System, TSE Systems GmbH, Bad-Homburg, Germany). The boxes were separated by a dark plexiglass wall and interconnected by an opening (3.5 × 10 cm) in the partition wall, thus allowing the animal to freely traverse from one compartment to the other. This wall divided the compartment into a dark (1 lx) and a brightly illuminated (100 lx) compartment. Each mouse was placed in the centre of the dark compartment to start the test. After a 5-s acclimatization period, the door automatically opened, allowing the animals free access to both the dark and bright compartments for 10 min. Innate anxiety was indexed and calculated based on the percent time spent in the light compartment during the 10 min of exploration ([time spent in light compartment/total time] × 100) and the latency until they moved to the light compartment as described before [87]. Total distance moved was measured to ascertain that percent time spent in the light compartment was not confounded by changes in the locomotor activity.

##### Open field test

Innate anxiety-like behaviour and locomotor activity were assed using the open field exploration test [49]. The open field exploration test was conducted in four identical square arenas (40 × 40 cm) surrounded by walls (35 cm high) as described in detail before [83, 88]. The apparatus was made of white Plexiglas and was located in a testing room under diffused lighting (28 lx in the centre zone, 20 lx in the corner of the maze). A digital camera was mounted directly above the four arenas, and images were captured using the EthoVision tracking system (Noldus Technology, Wageningen, Netherlands). The animals were gently placed in the centre of the arena and allowed to explore for the open field for 30 min. Innate anxiety-like behaviour was indexed by the time spent in the centre of the open field arena within the first 10 min. Locomotor activity was analysed by the total distance moved (cm) in the entire arena within 30 min.

#### Learned fear-related behaviour

##### Cued Pavlovian fear conditioning

Cued Pavlovian fear conditioning was conducted to assess differences in learned fear behaviour. The apparatus for fear conditioning (Multi Conditioning System, TSE System, Bad Hamburg, Germany) comprised 2 sets of test chambers to provide 2 distinct contexts (contexts A and B), both installed in ventilated, sound-insulated chests. The chambers of context A (30 length × 30 width × 36 height) were made of transparent acrylic glass and were equipped with a grid floor made of 29 stainless rods (4mm in diameter and 10 mm apart; inter-rod centre to inter-rod centre), through which a scrambled electric shock could be delivered. Scrambled foot shocks provided the unconditioned stimulus (US). The chambers of context B (30 length × 30 width × 36 height) were made of black acrylic glass and were equipped with a grey plastic floor instead of parallel grid floors. The chambers were equivalently illuminated by a house light (30 lx) and surrounded by 3 infrared light beam sensor system, with sensors spaced 14 mm apart, allowing movement detections in 3 dimensions. The cued Pavlovian fear conditioning consisted of 3 distinct phases, which were separated by 24 h each:

Conditioning (day 1): Following an initial habituation period of 6 min, the animals were exposed to 3 conditioning trials involving pairings between a conditioned stimulus (CS) and the US in context A. The CS was a 2.9-kHz tone measuring 58 dBA lasting for 30 s, which co-terminated with a 1-s, 0.3-mA foot shock US. The interval between each CS-US trial was 3 min. The amount of freezing between the three occasions of CS presentation provided a measure for the acquisition of fear conditioning. After conditioning, the animals were removed from the conditioning chambers and brought back to their home cages 3 min after the last foot shock.

Contextual fear expression (day 2): The expression of conditioned fear towards the context was assessed in the same context A without presenting the tone or shock. The time of freezing within this 6 min was evaluated and expressed as percent time freezing.

Cued conditioned fear expression (day 3): The expression of conditioned fear towards the CS was assessed in a novel context B. Following an initial acclimatization period of 3 min, the CS was delivered (without subsequent shock presentation) for 30 s for six times, each time followed by a break of 3min. During tone presentation, the time of conditioned freezing to the tone stimulus was evaluated and conditioned freezing was expressed as percent time freezing during the CS presentations.

##### Contextual fear expression and extinction

The apparatus and the setup to measure contextual fear conditioning are the same as for *cued Pavlovian fear conditioning* but with only one distinct context (context A). The test procedures consisted of three phases: habituation and conditioning, fear expression and fear extinction.

Habituation and conditioning: The animals were placed in the appropriate test chamber and were allowed to freely explore the chamber for 3 min. Conditioning commenced immediately at the end of the habituation period without the animals being removed from the chambers. Each trial began with a 1-s foot shock (US) set at 0.3 mA and was followed by a 90-s break period. The amount of freezing between the three occasions of US presentation provided a measure for the acquisition of fear conditioning. At the end of the 3 trials, the animals were removed from the boxes and were placed back in their home cages.

The fear expression phase took place 24 h later when the subjects were returned to the same chambers for a period of 6 min in the absence of any discrete stimulus.

The fear extinction phase was performed by returning the animals to the same chambers for a period of 6 min per day, without any discrete stimulus, for two more consecutive days.

The data on fear expressed as freezing behaviour collected in the distinct phases were analysed separately per day and expressed as percent time freezing.

Freezing was quantified automatically by program-guided algorithms as time of immobility.

### Molecular analyses

#### Collection of brain samples

After behavioural testing and a resting period of at least 7 days, the animals were deeply anaesthetized with an overdose of Pentobarbital (Streuli Pharma AG, Uznach, Switzerland) and perfused transcardially with 30ml phosphate-buffered saline (PBS, pH 7.4) at a flow rate of 20 ml/min. The brains were dissected, cut so that the ventricles were accessible, and immersion fixed in 50ml of 4% PFA for 6 h. After immersion fixation, the brains were cryoprotected in 30% sucrose in PBS, frozen with powdered dry ice and stored at −80°C until further processing.

#### Immunohistochemical analyses

Perfused brains were cut coronally at 30-μm thickness from frozen blocks with a sliding microtome. Eight serial sections were prepared for each animal and, after rinsing in PBS, stored at −20 °C in antifreeze solution (30% glycerol and 30% ethylene glycol in PBS at 25 mM and pH 7.4) until further processing. For immunohistochemical staining, the slices were first rinsed for 10 min in PBS. The sections were then incubated free-floating with the primary antibody overnight at 4°C under constant agitation (100 rpm). The following primary antibodies were used: rabbit anti-γH2AX (cell signalling 9718S; diluted 1:500 in 10 mM phosphate buffer pH 7.2 containing 4% normal goat serum (NGS)), rabbit anti-XRCC1 (Sigma HPA006717; diluted 1:400 in 10 mM phosphate buffer pH 7.2 containing 4% NGS), guinea pig anti-GABRA5 (in house made by Jean-Marc Fritschy laboratory UZH Zurich [89, 90]; diluted 1:1000 in PBS containing 0.2% Triton X-100 and 2% NGS), rabbit anti-GABRA1 (in house made by Jean. Marc Fritschy laboratory UZH Zurich [89, 90], diluted 1:8000 in PBS containing 0.2% Triton X-100 and 2% NGS) and rabbit anti-parvalbumin (cat. PV25, lot 84407422-05; diluted 1:1000 in PBS containing 0.2% Triton X-100 and 2% NGS).

Following incubation, the sections were washed three times with PBS (10 min each) and incubated with the secondary antibodies Cy3 diluted 1:300 and DAPI diluted 1:3000 in PBS containing 2% NGS for 30 min at room temperature and covered from light. The sections were washed again three times for 10 min in PBS, mounted and coverslipped with Dako fluorescence mounting medium (Cat# S302380-2, Agilent Technologies).

Immunofluorescence (IF)-stained images were acquired with a widefield microscope (AxioObserver Z1, Zeiss, Jena, Germany) using a 10× objective for XRCC1, 40× for γH2AX and 20× and 10× for GABAergic markers in the amygdala and hippocampus, respectively. Exposure times were set so that pixel brightness was never saturated and kept constant. For each animal, 3 images were acquired from 3 consecutive sections containing the dorsal hippocampus (Bregma −1.3 to −2.7 mm), ventral hippocampus (Bregma −2.9 to −3.5) and the amygdala (Bregma: −2.0 to −2.5). The images were analysed using NIH ImageJ software. The area of interest was delineated as shown in Figs. 5 and 6.

Quantification of XRCC1 immunoreactivity was performed by measuring mean staining intensity in the respective brain areas. For quantification of γH2AX foci, a number of foci present in 3 randomly selected areas of equal size in the areas of interest were counted. The mean value from the three areas was considered as one value for “number of foci per field”, as shown in the graph. Quantification of GABRA5 and GABRA1 immunoreactivity was achieved by measuring mean grey value. In brief, the brightness of the picture was set automatically and a background value of 200 pixels was subtracted from each image before the mean grey value was generated. The background-corrected relative optical densities were then averaged per animal. Parvalbumin-positive (PV+) cell numbers within the regions of interest were counted using the particle analyser plugin for the ImageJ software in the amygdala and were manually counted in the dorsal/ventral hippocampus. PV+ cell numbers were normalized to each individual area of interest and displayed as cells per square millimetre.

### Statistical analyses

Data for each sex were analysed separately, given existing evidence that similar molecular/genetic changes can induce distinct endophenotypes in males and female laboratory animals [91] and based on sex differences in the prevalence of neurodevelopmental and anxiety disorders [11, 38, 41]. All statistical analyses were performed using Prism (version 8, GraphPad Software, La Jolla, CA, USA), and data were analysed using parametric analysis of variance (ANOVA) or Student’s *t*-test. Immunohistochemical and behavioural data were analysed using Student’s *t*-test, with the exception of acquisition data for cued fear conditioning and contextual fear, which were analysed using a 3 × 2 (trial × genotype) repeated-measures ANOVA. Statistical significance was set at *p* < 0.05.

## Supplementary Information

**Additional file 1: Figure S1.** CamKIIa-Cre mediated XRCC1 KO in different regions of the forebrain. **Figure S2.** General locomotor activity and basal freezing levels during behavioral testing. **Figure S3.** Representative Images GABRA5 receptor density in XRCC1 male KO mice. **Figure S4.** GABRA1 receptor density in XRCC1 KO mice. **Figure S5.** Representative Images GABRA1 receptor density in XRCC1 male KO mice. **Figure S6.** Locomotor activity in CaMKIIa Cre transgene animals. **Table S1.** Cohorts of animals used for behavioral testing. **Table S2.** Summary of behavioral results. **Table S3.** Summary of molecular results.

## Data Availability

All data generated or analysed during this study are included in this published article [and its supplementary information files].
