# Supplementary material for: Deficient DNA base-excision repair in the forebrain leads to a sex-specific anxiety-like phenotype in mice
Source: BMC Biol. 2022 Jul 30;20:170. doi: 10.1186/s12915-022-01377-1 (PMC9339204; doi:10.1186/s12915-022-01377-1)
Supplement: Supplementary file 1 — Additional file 1: Figure S1. CamKIIa-Cre mediated XRCC1 KO in different regions of the forebrain. Figure S2. General locomotor activity and basal freezing levels during behavioral testing. Figure S3. Representative Images GABRA5 receptor density in XRCC1 male KO mice. Figure S4. GABRA1 receptor density in XRCC1 KO mice. Figure S5. Representative Images GABRA1 receptor density in XRCC1 male KO mice. Figure S6. Locomotor activity in CaMKIIa Cre transgene animals. Table S1. Cohorts of animals used for behavioral testing. Table S2. Summary of behavioral results. Table S3. Summary of molecular results. [file 12915_2022_1377_MOESM1_ESM.docx]

**Supplementary Information**

**Deficient DNA base-excision repair in the forebrain leads to a sex-specific anxiety-like phenotype in mice**

Flavia S. Mueller^1^, René Amport^1^, Tina Notter^2,3^, Sina M. Schalbetter^1^, Han-Yu Lin^1^, Zuzana Garajova^1^, Parisa Amini^1^, Ulrike Weber-Stadlbauer^1,2*§^, Enni Markkanen^1*§^

^1^ Institute of Veterinary Pharmacology and Toxicology, Vetsuisse Faculty, University of Zürich, 8057 Zürich, Switzerland

^2^ Neuroscience Center Zurich, University of Zurich and ETH Zurich, Zurich, Switzerland

^3^ Institute of Pharmacology and Toxicology, Faculty of Science, University of Zurich, 8057 Zurich, Switzerland

**Figure S1. CamKII-Cre mediated XRCC1 KO in different regions of the forebrain**. A) XRCC1 levels in the dorsal hippocampus of female mice. Representative images showing staining of brain sections of control (left) and XRCC1 KO (right) female animals with XRCC1 antibodies (top) and Dapi (bottom) with a focus on the hippocampus. B) Quantification of XRCC1 staining intensity as shown in A per area of interest in n = 3 per group. ** p<0.01 and *** p<0.001 based on independent Student’s t-test. Shown are all individual values, means ± SD. C) Quantification of XRCC1 staining intensity of brain sections of control and XRCC1 KO male animals with a focus on the ventral hippocampus. N=3 mice per group. Shown are all individual values, means ± SEM. D) Representative images showing staining of brain sections of different hippocampal areas in control (top) and XRCC1 KO (bottom) female animals with γH2AX antibodies (red) and Dapi (blue).

**Figure S2. General locomotor activity and basal freezing levels during behavioral testing.** Total distance moved in A) spontaneous alternation test, B) Y-maze spatial recognition memory test, C) social interaction test, D) light-dark box test and E) open-field test in male and female XRCC1 KO and control (CON) animals. Basal freezing levels of male and female XRCC1 KO and CON mice during the habituation phase in the F) cued pavlovian fear conditioning test and G) the contextual fear extinction test. B) G) N = 10 Con and 7 KO males, 9 Con and 5 KO females. A), C), D), E) and F) N = 5 Con and 6 KO males, 5 Con and 5 KO females. Shown are all individual values, means ± SEM.


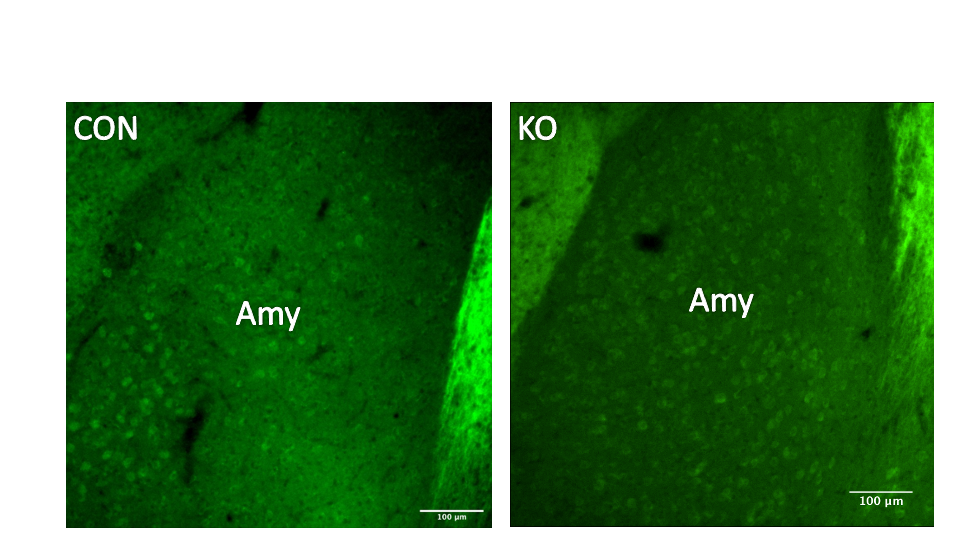


**Figure S3. GABRA5 receptor density in XRCC1 male KO mice.** The images show representative coronal brain sections of male control and XRCC1 KO animals stained with anti- GABRA5 antibody at the level of the Amygdala.


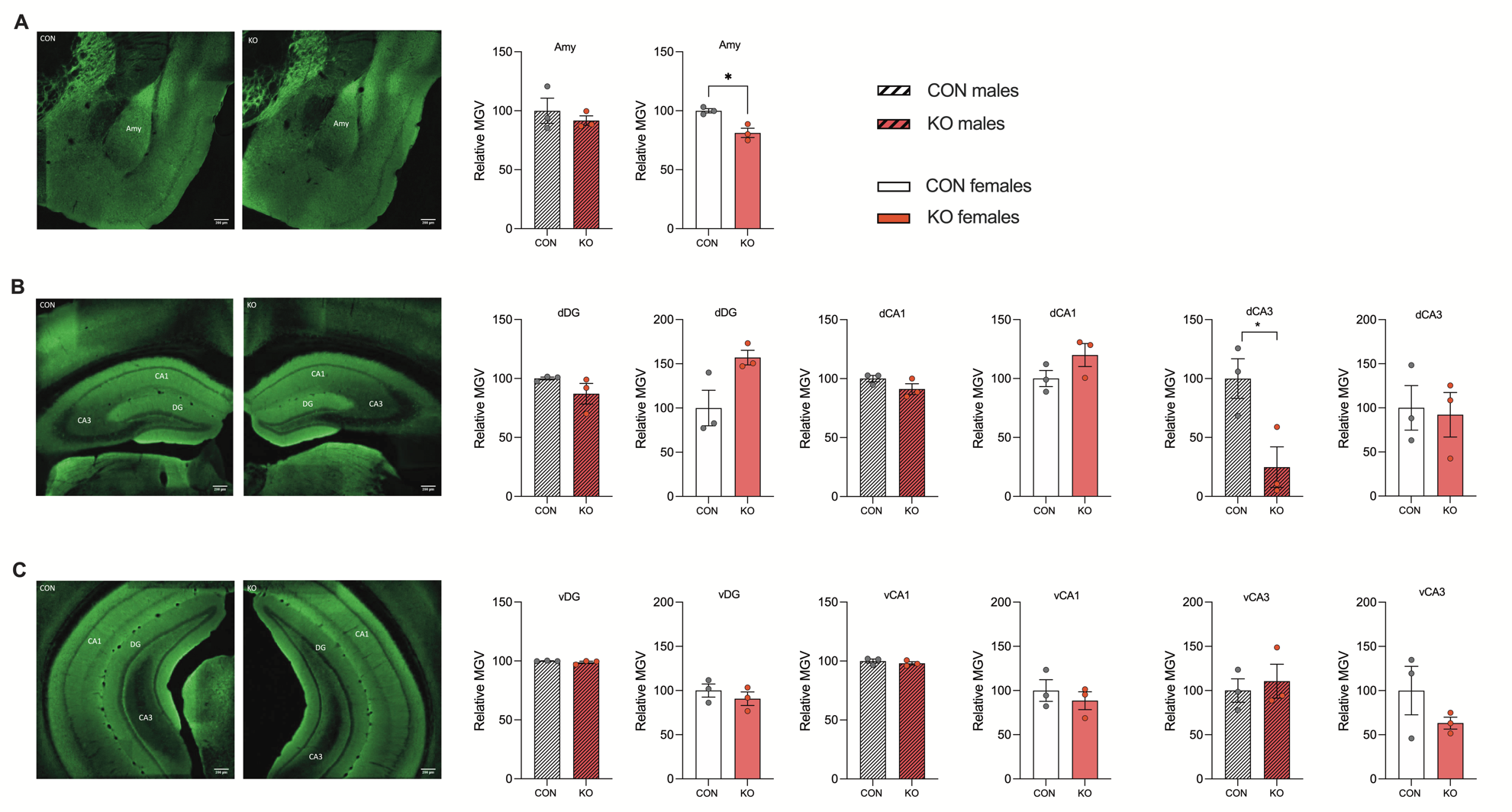


**Figure S4. GABRA1 receptor density in XRCC1 KO mice.** The images show representative coronal brain sections of female control and XRCC1 KO animals stained with anti- GABRA1 antibody at the level of A) the amygdala, B) the dorsal hippocampus and C) the ventral hippocampus. The bar plots depict the relative mean grey value (MGV) of GABRA1 in A) the amygdala (Amy), B) the dorsal and C) ventral DG, CA1 and CA3 (dDG, dCA1, dCA3 and vDG, vCA1, vCA3, respectively) of male and female XRCC1-KO and control mice. N=3 per sex and group. *p<0.05 based on independent Student’s t-test. Shown are all individual values, means ± SEM.


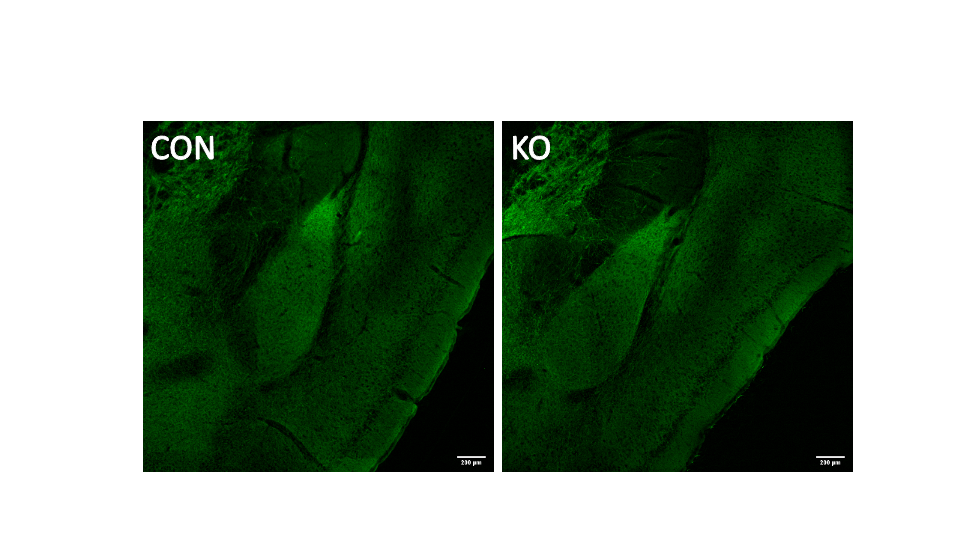


**Figure S5. GABRA1 receptor density in XRCC1 male KO mice.** The images show representative coronal brain sections of male control and XRCC1 KO animals stained with anti- GABRA1 antibody at the level of the Amygdala.

**
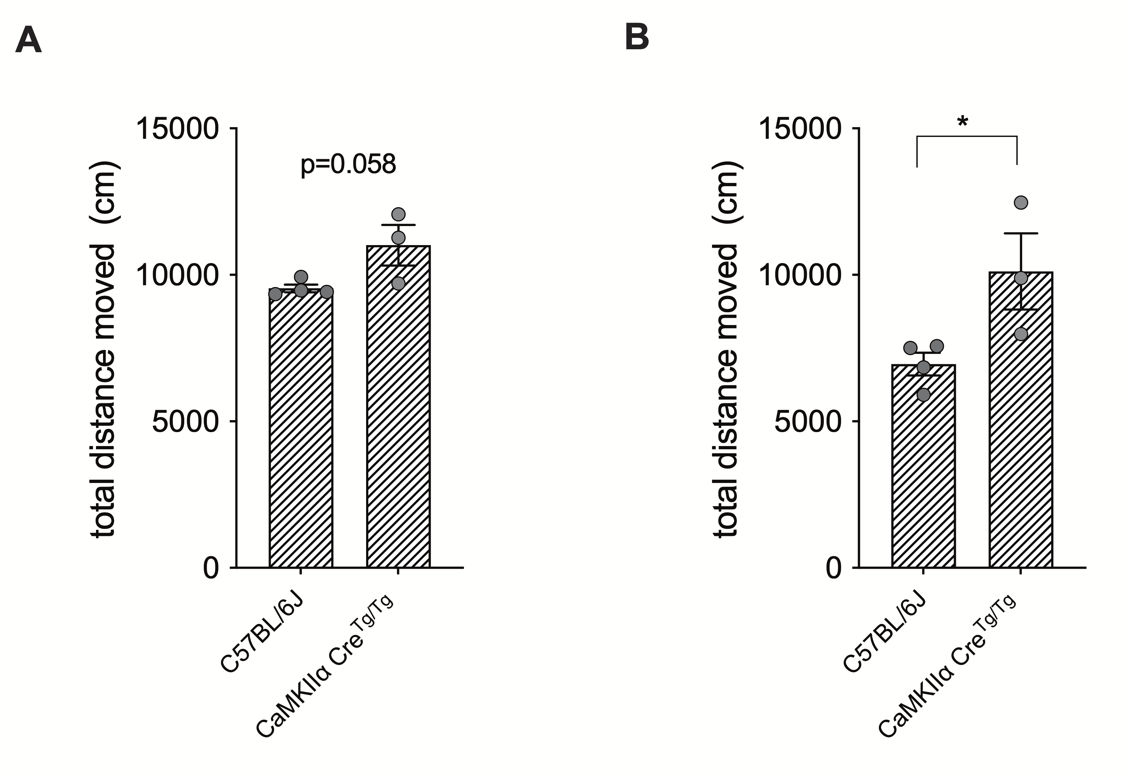
**

**Fig. S6. Locomotor activity in CaMKIIα Cre transgene animals**. A) Light-dark box test. The bar plots depict total distance moved in cm for CaMKIIα Cre^Tg/Tg^ transgene animals compared to C57BL/6J animals. B) Open field test. Total distance moved (cm) by CaMKIIα Cre^Tg/Tg^ and C57BL/6J animals. *p<0.05 based on Student’s t-test. N = 4 C57BL/6J and 3 CaMKIIα Cre^Tg/Tg^ male animals. Shown are all individual values, means ± SEM.

| **Cohort** | **Genotype** | **Test** | **Number of animals** |
| --- | --- | --- | --- |
| **I** | C57BL/6J | LD, OF | 4m |
|  | CaMKIIa Cre^Tg/Tg^ |  | 3m |
| **II** | CON | Rotarod, LD, OF, SA, SI, FC | 5m, 5f |
|  | KO |  | 6m, 5f |
| **III** | CON | Y-maze, CF | 10m, 9f |
|  | KO |  | 7m, 5f |

**Table S1. Cohorts of animals used for behavioral testing.** Three independent cohorts were generated and used for the herein presented work. For each cohort, the table summarizes the number of animals per sex and their allocation to the experiments of interest. Whereas cohort 1 was used to determine potential cofounding effects of the expression of Cre on behavior, cohort 2 and 3 were used for specific behavioral phenotyping. In cohort 3, a subcohort (N = 5 per sex and group) was used for immunohistochemical investigations of GABRA5 and PV and a subcohort (N = 3 per sex and group) for immunohistochemical investigations of GABRA1. In cohort 2, a subcohort was used to confirm XRCC1 KO (N = 3 per group and sex) and a subcohort (N = 3) to confirm presence of DNA damage. m, males; f, females. LD, light-dark box test; OF, open-field test, SA, spontaneous alternation test; SI, social interaction test; FC, cued pavlovian fear conditioning test; CF, contextual fear extinction test.

| Test | Male KO vs. CON | Female KO vs. CON |
| --- | --- | --- |
| Body Weight | | |
| Body Weight (g) | *= (0.99)* | *= (0.62)* |
| Motor Coordination | | |
| Rotarod | | |
| Time on Rotarod (s) | *= (0.33)* | *= (0.44)* |
| Cognition | | |
| Spontaneous Alternation Test | | |
| % alternation | *= (0.45)* | *= (0.34)* |
| Y-Maze | | |
| % time novel arm | *= (0.24)* | *= (0.65)* |
| Social Behavior | | |
| Social Interaction Test | | |
| % time mouse | *= (0.31)* | *= (0.96)* |
| Anxiety | | |
| Light-Dark-Box | | |
| % time in bright | ***↓ (*0.024)*** | *= (0.34)* |
| Latency (s) | *= (0.15)* | *= (0.31)* |
| Open Field | | |
| time in CZ (s) | ***↓ (**0.001)*** | *= (0.31)* |
| Learned Fear | | |
| Cued Pavlovian Fear Conditioning | | |
| Acquisition | *= (0.37)* | ***↑ (****0.0003)*** |
| Contextual Fear Expression | *= (0.64)* | ***↑ (*0.019)*** |
| CS-cued Fear Expression | *= (0.96)* | *= (0.14)* |
| Contextual Fear Extinction Test | | |
| Acquisition | *= (0.39)* | *= (0.50)* |
| Contextual Fear Expression D2 | *= (0.24)* | ***↑ (0.059)*** |
| Contextual Fear Expression D3 | *= (0.75)* | *= (0.21)* |
| Contextual Fear Expression D4 | *= (0.39)* | *= (0.23)* |

**Table S2. Summary of behavioral results.** = no change compared to CON, ↑ Increased measure compared to CON, ↓ Decreased measure compared to CON. Values in brackets index p-values of t-tests or main effects of genotype for ANOVAs (acquisition phases).

| Molecular Marker | Male KO vs. CON | Female KO vs. CON |
| --- | --- | --- |
| XRCC1 | | |
| dDG | *↓ (*****0.0004****)* | *↓ (*****0.021****)* |
| dCA1 | *↓* ***(*0.0002****)* | *↓ (*****0.0006****)* |
| dCA2 | *↓ (*****0.0004****)* | *↓ (*****0.0279****)* |
| dCA3 | *= (0.1401)* | *= (0.061)* |
| Amy | *↓ (*****0.0079****)* | *↓ (*****0.0125****)* |
| *γH2AX* | | |
| dDG | *↑ (*****0.0008****)* | *NA* |
| dCA1 | *↑ (*****0.0021****)* | *NA* |
| dCA2 | *↑ (*****0.0278****)* | *NA* |
| dCA3 | *= (0.081)* | *NA* |
| Amy | *= (0.2746)* | *NA* |
| GABRA5 | | |
| dDG | *= (0.11)* | *= (0.11)* |
| dCA1 | *= (0.12)* | ***↓ (*0.012)*** |
| dCA3 | ***↓ (*0.019)*** | *= (0.96)* |
| vDG | *= (0.98)* | *= (0.87)* |
| vCA1 | *= (0.09)* | *= (0.89)* |
| vCA3 | *= (0.89)* | *= (0.29)* |
| Amy | *= (0.37)* | ***↓ (**0.009)*** |
| PV | | |
| dDG | *= (0.94)* | *= (0.86)* |
| dCA1 | *= (0.21)* | *= (0.42)* |
| dCA3 | *= (0.71)* | *= (0.50)* |
| vDG | ***↑ (**0.007)*** | *= (0.30)* |
| vCA1 | *= (0.23)* | *= (0.84)* |
| vCA3 | *= (0.31)* | *= (0.85)* |
| Amy | *= (0.27)* | *= (0.65)* |

**Table S3. Summary of molecular results.** NA not applicable, readout not assessed in that group, = no change compared to CON, ↑ Increased measure compared to CON, ↓ Decreased measure compared to CON. Values in brackets index p-values of t-tests.
